# Occurrence of *Clostridium perfringens* and Molecular Detection of *Clostridioides difficile* Toxin-Gene Targets in Foal Fecal Samples: A Multiregional Study in Türkiye

**DOI:** 10.3390/vetsci13101060

**Published:** 2026-10-09

**Authors:** Burcu Karagülle, Zeynep Yerlikaya

**Affiliations:** Department of Microbiology, Faculty of Veterinary Medicine, Fırat University, 23200 Elazığ, Türkiye; zyerlikaya@firat.edu.tr

**Keywords:** foal, *Clostridium perfringens*, *Clostridioides difficile*, fecal occurrence, toxin genes, molecular detection, Türkiye

## Abstract

*Clostridium perfringens* and *Clostridioides difficile* are associated with equine enterocolitis, but data on foals in Türkiye are limited. This multiregional study examined 300 archived fecal samples from 2–6-month-old foals originating from 20 stud farms in five provinces. Gastrointestinal signs were recorded in ten foals; systematic clinical information was unavailable for 290. *Clostridium perfringens* was investigated by anaerobic culture followed by PCR confirmation and toxin-gene profiling (*cpa*, *cpb*, *cpb2*, *etx*, *iap*, *cpe*, and *netB*). *Clostridioides difficile*-specific DNA was investigated by PCR of DNA extracted from archived fecal material, targeting *tpi*, *tcdA*, and *tcdB*. *Clostridium perfringens* was confirmed in 50/300 samples (16.7%; 95% CI, 12.9–21.3%); confirmed isolates had a *cpa*-only profile among the genes investigated. The *tpi* target was detected in 12/300 samples (4.0%; 95% CI, 2.3–6.9%), of which seven also contained *tcdA* and *tcdB* (2.3% overall; 95% CI, 1.1–4.7%). *cpa*-confirmed *C. perfringens* and the *C. difficile*-specific *tpi* target was detected in samples from all five provinces; geographic comparisons were descriptive and exploratory. Because *C. difficile* was not cultured and toxin production was not assessed, the results represent species-specific and toxin-gene targets rather than viable toxigenic isolates or active disease. Clinical associations could not be evaluated. These findings provide multiregional baseline data and support prospective studies incorporating clinical assessment, culture, quantitative testing, and toxin detection.

## Simple Summary

Two spore-forming bacteria, *Clostridium perfringens* and *Clostridioides difficile*, may be detected in the equine intestine and are associated with diarrhea and enterocolitis, particularly in young foals. We examined 300 archived fecal samples from 2–6-month-old foals originating from 20 stud farms in five provinces of Türkiye. Gastrointestinal signs were recorded in ten foals, whereas systematic clinical information was unavailable for the remaining animals. *Clostridium perfringens* was molecularly confirmed in 50 samples (16.7%; 95% CI, 12.9–21.3%); confirmed isolates showed a *cpa*-only toxin-gene profile among the genes investigated. *Clostridioides difficile*-specific *tpi* DNA was detected in 12 samples (4.0%; 95% CI, 2.3–6.9%), and seven samples also contained both *tcdA* and *tcdB* targets (2.3%; 95% CI, 1.1–4.7%). Both bacterial targets were detected in samples from all five provinces. Because the *C. difficile* investigation was molecular and did not include culture or toxin testing, the findings indicate bacterial and toxin-gene DNA rather than viable organisms, toxin production, or clinical disease. The clinical observations remain descriptive. This study provides new multiregional baseline data for Turkish foals and supports prospective investigations combining standardized clinical assessment, culture, quantitative methods, and toxin detection.

## 1. Introduction

Clostridial diseases of horses encompass enteric/enterotoxic, histotoxic, and neurotoxic syndromes. Among the enteric conditions, those associated with *Clostridium perfringens* and *Clostridioides difficile* are clinically important because they may present as diarrhea, enterocolitis, or rapidly progressive systemic illness, particularly in foals [1]. However, diagnostic interpretation is challenging: both organisms may be detected in clinically affected animals, but colonization or fecal shedding can also occur without toxin-mediated disease [1,2,3,4]. Consequently, organism detection, toxin-gene carriage, toxin production, and clinical disease should be regarded as related but distinct findings.

*Clostridium perfringens* is an anaerobic, spore-forming bacterium with a broad environmental distribution and an extensive toxin repertoire. The revised toxinotyping scheme classifies strains into types A–G based on the presence of genes encoding alpha (*cpa*/*plc*), beta (*cpb*), epsilon (*etx*), iota (*iap*/*ibp*), enterotoxin (*cpe*), and NetB (*netB*) toxins [5]. The beta2-toxin gene (*cpb2*) is not a defining component of this scheme but has been extensively studied in animal enteric disease. Because *cpa* is present in all recognized toxinotypes, its detection supports molecular confirmation of *C. perfringens* but, by itself, provides limited information about enteric virulence [1,5]. Broader toxin-gene profiling is therefore needed to distinguish *cpa*-only strains from isolates carrying toxin determinants with greater disease specificity.

*Clostridioides difficile* is an anaerobic, spore-forming bacterium capable of causing toxin-mediated diarrhea and colitis in horses [1,4]. Toxins A and B, encoded by *tcdA* and *tcdB*, are the principal virulence factors assessed in routine molecular characterization. The triose phosphate isomerase gene (*tpi*) provides a species-specific PCR target, and its combined detection with *tcdA* and *tcdB* enables simultaneous identification and toxin-gene profiling [6]. Classical equine studies support an association between toxin detection and intestinal disease. Donaldson and Palmer detected *C. difficile* toxin A, *C. perfringens* enterotoxin, or both, in diarrheic horses, whereas neither toxin was detected in the corresponding non-gastrointestinal control group [7]. Madewell et al. documented toxigenic *C. difficile* in nine of ten horses during apparent outbreaks of acute diarrhea in a veterinary teaching hospital, compared with one of 23 hospitalized horses without diarrhea [8]. Severe and fatal equine colitis has also been associated with epidemic *C. difficile* lineages, including NAP1/027 [9].

However, detecting *C. difficile* does not equate to clinical infection. Culture-based studies have recovered toxigenic strains from clinically healthy and affected horses, and reported occurrence varies with age, management, hospitalization, antimicrobial exposure, and diagnostic method [4,10,11,12]. Results obtained by culture, PCR testing of fecal DNA, toxin immunoassay, and cytotoxicity testing therefore require method-specific interpretation.

Despite the clinical and epidemiological importance of *C. perfringens* and *C. difficile*, studies evaluating both organisms in the same equine population remain limited. In Türkiye, *C. difficile* has been investigated in cattle feces, carcasses, and slaughterhouse environments [13], whereas *C. perfringens* has been characterized in broiler chickens [14] and in small ruminants with hemorrhagic abomasitis [15]. A recent retrospective survey also described bacteria recovered from equine clinical submissions but did not conduct targeted anaerobic surveillance or concurrent species-specific and toxin-gene testing for these two organisms [16]. Thus, the specific gap is the concurrent investigation of these organisms in fecal samples from a multiregional foal population, rather than a complete absence of Turkish data on either organism.

The objectives of the present study were (i) to describe the sample-level occurrence and geographic distribution of *cpa*-confirmed *C. perfringens* and *C. difficile*-specific DNA and toxin-gene targets in archived fecal samples from 2–6-month-old foals originating from five provinces of Türkiye and (ii) to characterize the toxin-gene profiles detected through culture-based isolation of *C. perfringens* and PCR screening of DNA extracted from archived fecal material for *C. difficile*-specific and toxin-gene targets. The study was not designed to estimate population prevalence, establish disease causation, or infer farm- or province-level risk.

## 2. Materials and Methods

### 2.1. Study Design and Sample Origin

This study examined 300 archived fecal samples from a previously described field collection comprising 428 foal samples from 20 stud farms in five provinces of Türkiye [17]. Samples with sufficient residual material and preserved container integrity were included on the basis of suitability for the planned laboratory analyses; selection was not stratified by province, clinical information, or previous microbiological findings. The analyzed samples originated from Malatya (*n* = 23; one farm), Şanlıurfa (*n* = 44; three farms), Bursa (*n* = 71; three farms), Eskişehir (*n* = 100; five farms), and İzmit/Kocaeli (*n* = 62; eight farms). The numbers of analyzed samples contributed by the individual farms were as follows: Malatya, A1 (*n* = 23); Şanlıurfa, B1 (*n* = 23), C1 (*n* = 4), and C2 (*n* = 17); Bursa, A3 (*n* = 31), B2 (*n* = 23), and C3 (*n* = 17); Eskişehir, A2 (*n* = 36), B3 (*n* = 42), C4 (*n* = 5), C5 (*n* = 5), and C6 (*n* = 12); and İzmit/Kocaeli, B4 (*n* = 35), C7 (*n* = 1), C8 (*n* = 2), C9 (*n* = 5), C10 (*n* = 2), C11 (*n* = 12), C12 (*n* = 2), and C13 (*n* = 3). The participating farms comprised seven institutional and 13 private premises. Foals were 2–6 months old. Gastrointestinal signs were recorded in ten foals, including four with diarrhea and six with colic. The clinical status of the remaining 290 foals could not be verified retrospectively; therefore, the overall study population was not classified as clinically healthy or diseased.

### 2.2. Ethical Approval

The use of archived equine fecal samples and the laboratory analyses conducted in the present study were approved by the Fırat University Local Ethics Committee for Animal Experiments on 10 June 2026 (decision/session no. 2026/12-07). The samples originated from a previously approved field collection (protocol no. 2016/129), described elsewhere [17].

### 2.3. Sample Collection, Transport, and Storage

The original fecal samples were collected from 1 April to 1 June 2017 by rectal swabbing with sterile charcoal Amies transport swabs (LP Italiana, Milan, Italy) and transported under cold-chain conditions [17]. Fecal material was transferred to Cooked Meat Medium containing 50% glycerol and stored continuously at −86 °C for approximately nine years. Because all samples were collected during the same two-month field period and maintained under the same storage conditions until analysis in 2026, storage duration was broadly comparable among farms and provinces. The archived preparations had not been thawed previously. Each selected tube was thawed once in 2026, and material from the same thaw event was used concurrently for *C. perfringens* culture and *C. difficile* molecular analysis.

### 2.4. Isolation and Phenotypic Identification of C. perfringens

For isolation of *C. perfringens*, material from the thawed archived preparations was inoculated into fresh Cooked Meat Medium (Oxoid, Basingstoke, UK) and incubated anaerobically at 37 °C for 48 h. Enriched cultures were streaked onto tryptose sulfite cycloserine agar (Merck, Darmstadt, Germany) supplemented with D-cycloserine and incubated anaerobically at 37 °C for 24 h using Anaerocult^®^ A (Merck). From each culture showing presumptive growth, at least five well-isolated black colonies were transferred separately to 5% sheep blood agar. Following purification and phenotypic evaluation, three single colonies were randomly selected from each presumptive-positive sample to assess potential within-sample variation. DNA was extracted separately from each selected colony, and species confirmation and toxin-gene profiling were performed independently; colonies were not pooled. The selected colonies met the same presumptive phenotypic criteria. Distinct colony morphotypes were not categorized separately in the archived laboratory records; therefore, no morphotype-specific comparison was performed.

### 2.5. DNA Extraction and PCR Toxinotyping of C. perfringens

DNA was extracted separately from purified single-colony cultures using the DNeasy UltraClean Microbial Kit (QIAGEN, Hilden, Germany) according to the manufacturer’s instructions. Molecular confirmation and toxin-gene profiling targeted *cpa*, *cpb*, *cpb2*, *etx*, *iap*, *cpe*, and *netB*. The panel reflected the assays available in the laboratory and was used to explore the investigated toxin-gene profiles; *netB* was retained because it had previously been screened in an equine *C. perfringens* investigation, whereas *netF* was not part of the available panel. Accordingly, toxin-profile interpretations were restricted to the genes investigated.

For *cpa* PCR, each 50-µL reaction contained 5 µL of 10× PCR buffer, MgCl2 at a final concentration of 2.5 mM, each dNTP at 250 µM, each primer at 0.2 µM, 5 U of Taq DNA polymerase, and 5 µL of template DNA. Cycling consisted of 94 °C for 5 min; 30 cycles of 94 °C for 1 min, 55 °C for 1 min, and 72 °C for 1 min; and 72 °C for 3 min. The multiplex assay for *cpa*, *cpb*, *cpb2*, *etx*, *iap*, and *cpe* was performed in 50 µL containing 6 µL of 10× PCR buffer, MgCl2 at 3.0 mM, each dNTP at 250 µM, each primer at 0.2 µM, 5 U of Taq DNA polymerase, and 5 µL of template DNA. Cycling consisted of 94 °C for 2 min; 30 cycles of 94 °C for 1 min, 55 °C for 1 min, and 72 °C for 2 min; and 72 °C for 5 min. The separate *netB* reaction used the *cpa* reaction composition and cycling at 94 °C for 2 min; 35 cycles of 94 °C for 30 s, 55 °C for 30 s, and 72 °C for 1 min; and 72 °C for 12 min. Reference strains NCTC 13110 (ATCC 3626), NCTC 8239, and CCUG 44727 were included as positive controls for the multiplex toxin-gene assays. A *C. perfringens* type A reference strain from the Pendik Veterinary Control Institute was used for *cpa*, and DNA from the *netB*-positive EHE-NE18 strain supplied by Prof. Julian Rood (Monash University, Australia) was used for *netB*. Distilled water served as the negative control. Amplicons were separated in 1.5% agarose gels alongside a 100-bp DNA ladder and visualized after ethidium-bromide staining under UV transillumination. A reaction was considered positive when a band of the expected size was present in the sample and positive-control lanes and absent from the negative-control lane.

### 2.6. DNA Extraction from Archived Fecal Material for C. difficile-Specific DNA Detection

DNA was extracted from archived equine fecal material using the QIAamp PowerFecal Pro DNA Kit (QIAGEN, Hilden, Germany). A 250-µL aliquot of the thawed fecal-glycerol suspension in Cooked Meat Medium was centrifuged at 10,000× *g* for 5 min, and the supernatant was discarded. The pellet was resuspended in the kit lysis buffer, transferred to PowerBead tubes, and homogenized for 10 min at maximum speed; subsequent purification followed the manufacturer’s protocol. The *C. difficile* component was predefined as a molecular investigation because prolonged storage was expected to limit reliable recovery of viable organisms; culture- and toxin-production assays were therefore outside the study scope.

### 2.7. PCR Detection of C. difficile-Specific DNA and Toxin-Gene Targets

DNA extracted from the archived fecal material was screened for the *C. difficile*-specific *tpi* gene and the toxin genes *tcdA* and *tcdB*. Primers described by Lemée et al. [6] were used with reaction and cycling conditions adapted from Abay et al. [13]. The *tpi* target was amplified separately, whereas *tcdA* and *tcdB* were examined by multiplex PCR. Primer sequences and expected amplicon sizes are provided in Table 1.

PCR was performed in a total volume of 25 µL. Each reaction contained 2.5 µL of 10× PCR buffer (50 mM Tris-HCl, 10 mM KCl), 0.5 µL of 10 mM dNTP mix, 3 µL of MgCl_2_, 0.5 µL of DreamTaq Hot Start DNA Polymerase, each primer at 0.4 µM, and 2.5 µL of template DNA; nuclease-free water was added to reach the final volume. Amplification consisted of 94 °C for 10 min; 35 cycles of 94 °C for 50 s, 54 °C for 40 s, and 72 °C for 50 s; and 72 °C for 10 min. DNA extracted from a previously characterized *tpi*-, *tcdA*-, and *tcdB*-positive *C. difficile* isolate was used as the positive amplification control for all three targets; the control DNA was provided by Prof. Dr. Seçil Abay, Erciyes University, Kayseri, Türkiye. Nuclease-free water served as the negative control. An internal extraction/amplification control was not included.

### 2.8. Statistical Analysis

Descriptive statistics summarized sample-level findings. Exact numerators and denominators, percentages, and two-sided 95% Wilson confidence intervals were calculated for the overall and province-specific occurrence estimates. Because the archived specimens were clustered within farms, farm sizes and provincial sample numbers were unequal, and positive *C. difficile* counts were small, province-level comparisons were treated as exploratory and presented descriptively; no inference of equal or uniform occurrence was made. No adjustment for farm-level clustering was performed. Statistical summaries were prepared using SPSS software (version 22.0; IBM Corp., Armonk, NY, USA).

## 3. Results

### 3.1. Geographic Distribution of the Examined Samples

A total of 300 archived foal fecal samples were examined: Eskişehir, 100 (33.3%); Bursa, 71 (23.7%); İzmit/Kocaeli, 62 (20.7%); Şanlıurfa, 44 (14.7%); and Malatya, 23 (7.7%). The samples originated from 20 farms across the five provinces. Sample-level culture and molecular findings, with 95% confidence intervals, are summarized in Table 2.

### 3.2. Occurrence of C. perfringens

Presumptive *C. perfringens* growth was obtained from 53 of 300 samples (17.7%). Three single-colony isolates from each presumptive-positive sample were examined independently. All three colonies from each of 50 samples were *cpa*-positive, confirming *C. perfringens* in 50/300 samples (16.7%; 95% CI, 12.9–21.3%). All three colonies from each of the remaining three presumptive-positive samples were *cpa*-negative; these samples originated from Malatya, Şanlıurfa, and Eskişehir. No discordance was observed among the three colonies examined within any sample.

*cpa*-confirmed *C. perfringens* was detected in samples from all five provinces: Malatya, 4/23 (17.4%; 95% CI, 7.0–37.1%); Şanlıurfa, 7/44 (15.9%; 95% CI, 7.9–29.4%); Bursa, 12/71 (16.9%; 95% CI, 9.9–27.3%); Eskişehir, 17/100 (17.0%; 95% CI, 10.9–25.5%); and İzmit/Kocaeli, 10/62 (16.1%; 95% CI, 9.0–27.2%). These geographic estimates are descriptive and exploratory.

### 3.3. Toxin-Gene Findings in cpa-Confirmed C. perfringens Isolates

All *cpa*-positive single-colony isolates recovered from the 50 confirmed samples were negative for *cpb*, *cpb2*, *etx*, *iap*, *cpe*, and *netB*. Thus, the three colonies examined from each confirmed sample showed a concordant *cpa*-only toxin-gene profile among the genes investigated. This profile is compatible with toxin type A within the limits of the tested panel.

### 3.4. PCR Detection of C. difficile-Specific DNA

The *C. difficile*-specific *tpi* target was detected in 12 of 300 archived fecal samples (4.0%; 95% CI, 2.3–6.9%). Positive samples occurred in all provinces: Malatya, 1/23 (4.3%; 95% CI, 0.8–21.0%); Şanlıurfa, 2/44 (4.5%; 95% CI, 1.3–15.1%); Bursa, 2/71 (2.8%; 95% CI, 0.8–9.7%); Eskişehir, 5/100 (5.0%; 95% CI, 2.2–11.2%); and İzmit/Kocaeli, 2/62 (3.2%; 95% CI, 0.9–11.0%). These estimates are descriptive and exploratory.

Of the 12 *tpi*-positive samples, seven also contained *tcdA* and *tcdB* targets (58.3% of *tpi*-positive samples; 2.3% overall; 95% CI, 1.1–4.7%). The remaining five were negative for both toxin-gene targets. Because the assays were performed on total DNA extracted from archived fecal material without *C. difficile* culture or toxin testing, these findings indicate target co-detection and do not establish viable organisms, toxin production, or that all targets originated from the same viable strain.

### 3.5. Findings in Foals with Recorded Gastrointestinal Signs

Recorded gastrointestinal signs were available for ten foals: four with diarrhea and six with colic. All ten samples were positive for *cpa*-confirmed *C. perfringens*. The *tpi*, *tcdA*, and *tcdB* targets were co-detected in one diarrheic foal and three foals with colic. Individual age, farm of origin, antimicrobial exposure, and additional clinical history were not available in the archived records for these ten foals. These observations are strictly descriptive; because clinical information was unavailable for the remaining 290 foals and no systematically characterized unaffected comparison group was available, the study cannot estimate an association between the detected targets and gastrointestinal disease.

## 4. Discussion

### 4.1. Principal Findings and Study Context

This study provides a multiregional assessment of *cpa*-confirmed *C. perfringens* and *C. difficile*-specific DNA targets in archived fecal samples from foals originating from 20 farms in five provinces of Türkiye. *cpa*-confirmed *C. perfringens* occurred in 16.7% of samples, and the examined colonies showed a *cpa*-only profile among the genes investigated. The *C. difficile*-specific *tpi* target occurred in 4.0% of samples, including seven samples with co-detection of *tcdA* and *tcdB*. Both targets were detected in samples from every province; however, geographic estimates were exploratory and do not demonstrate uniform occurrence.

Clinical observations were available for only ten foals. Although all ten yielded *cpa*-confirmed *C. perfringens* and four also contained *tpi*, *tcdA*, and *tcdB* targets, the absence of systematic clinical information for the remaining population precluded comparison with a verified unaffected group. Neither bacterial load nor toxin production was assessed. The clinical findings are therefore descriptive and hypothesis-generating and do not support causal or disease-association inferences.

Previous Turkish studies have investigated related clostridial organisms in cattle, slaughterhouse environments, poultry, small ruminants, and equine clinical submissions [13,14,15,16,20]. These studies differ in host species, clinical selection, sample matrix, and laboratory method and therefore provide context rather than direct comparators. Broader studies further show that sampling point and production-chain context can strongly influence detection and interpretation [21,22]. The specific contribution of the present study is the concurrent investigation of *cpa*-confirmed *C. perfringens* and *C. difficile*-specific DNA and toxin-gene targets in archived fecal samples from the same multiregional foal population.

### 4.2. Comparison with Previous Equine Studies

The sample-level estimates observed here—16.7% for *cpa*-confirmed *C. perfringens* and 4.0% for the *C. difficile*-specific *tpi* target—fall within the broad range reported in equine investigations. Direct comparisons remain limited by differences in animal age, clinical status, management, sample storage, and diagnostic method [23,24,25]. This is particularly relevant because *C. perfringens* was assessed by culture and isolate-level PCR, whereas *C. difficile* was assessed molecularly in DNA extracted from archived fecal material.

The 16.7% occurrence of *cpa*-confirmed *C. perfringens* was numerically similar to frequencies reported in equine studies from South Korea and Brazil [2,26]. However, those populations differed in clinical selection, age, and sampling context. Substantially higher recovery has been reported during the first days of life [3]. Other equine reports have documented type C disease, coinfection, and diverse toxin-associated profiles [27,28,29,30]. The lower frequency in the present 2–6-month-old cohort may partly reflect age, although culture protocols, archived storage, geography, and farm management also differ.

For *C. difficile*, the 4.0% *tpi*-target detection estimate was numerically close to the 2.4% culture-based occurrence reported in a South Korean survey [31]. However, culture followed by isolate characterization differs fundamentally from PCR testing of DNA extracted from archived fecal material. Higher frequencies have generally been reported in neonatal or hospitalized populations [24,25,32,33,34,35,36,37]. Age, hospitalization, antimicrobial exposure, environmental spore burden, clinical selection, and diagnostic method can all affect detection. The comparatively low estimate in the present older, field-derived cohort cannot be attributed to a single factor because complete clinical, exposure, and environmental data were unavailable.

### 4.3. Molecular Interpretation of C. difficile and Its Toxin-Gene Targets

PCR detection in DNA extracted from archived fecal material requires careful interpretation. Detection of *tpi* indicates *C. difficile*-specific DNA but does not confirm viable organisms or active shedding. Likewise, *tcdA* and *tcdB* detection establishes the presence of toxin-gene targets, not toxin expression or their origin from the same viable strain. The seven positive samples are therefore described as *tpi*/*tcdA*/*tcdB* target co-detection. Culture, isolate characterization, and toxin assays would be required to establish viability, strain genotype, and toxin production.

### 4.4. Toxin-Gene Profiles of C. perfringens

All *cpa*-positive isolates recovered from the 50 confirmed samples displayed a *cpa*-only profile among the seven genes investigated. This profile is compatible with toxin type A within the limits of the panel, rather than definitive comprehensive toxin typing, because *cpa* occurs in every recognized toxin type. Three independently tested colonies per sample yielded concordant profiles, although testing a finite number of colonies cannot exclude unobserved within-sample strain diversity. The absence of the additional genes examined does not establish absence of virulence or toxin production. This profile differs from the *cpb2*-positive strains previously reported in horses with intestinal disease [38].

The selected panel reflected assays available in the laboratory and included *netB* partly because this gene had previously been screened in an equine *C. perfringens* investigation. Although *netB* is principally associated with avian necrotic enteritis, its inclusion allowed exploratory assessment in this archived foal material. The equine-relevant *netF* gene was not included; therefore, no conclusion can be drawn regarding *netF* occurrence. This is important because NetF-producing strains have been associated with necrotizing enteritis in neonatal foals [39,40,41,42].

### 4.5. Geographic Distribution and One Health Considerations

*cpa*-confirmed *C. perfringens* and the *C. difficile*-specific *tpi* target were detected in samples from all five provinces. Province-specific confidence intervals were wide and overlapping, farm and sample numbers varied, positive *C. difficile* counts were small, and clustering within farms was not modeled. The geographic findings are therefore descriptive and exploratory; these findings do not establish equal or uniform occurrence among provinces.

The detection of these organisms or genetic targets in equine fecal material may warrant future One Health investigation because clostridial spores can persist in animal-associated environments and related *C. difficile* lineages have been reported across equine, human, and environmental sources [11,43]. However, the present study did not investigate transmission, environmental sources, or genomic relatedness and did not culture *C. difficile*. No inference regarding viable reservoirs or zoonotic transmission is therefore made.

### 4.6. Study Strengths and Limitations

Study strengths include examination of 300 archived samples from 20 farms across five provinces, concurrent assessment of two clinically relevant clostridial targets in the same sample population, independent testing of three *C. perfringens* colonies per presumptive-positive sample, and complementary culture-based and molecular approaches. The study provides baseline sample-level data for an underrepresented geographic and host population.

The findings should be interpreted within the study design. Inclusion depended on the availability and integrity of archived material, so the estimates describe the analyzed sample set rather than population prevalence. The specimens had been stored at −86 °C in Cooked Meat Medium with 50% glycerol for approximately nine years and were thawed once; prolonged storage may have reduced culturability, although molecular targets may persist when organisms are no longer viable. *C. difficile* was assessed without culture, toxin testing, ribotyping, or sequencing, and no internal extraction/amplification control was used; therefore, inhibition or reduced extraction efficiency in the archived fecal matrix cannot be excluded. The *C. perfringens* analysis did not quantify bacterial load or toxin production, and *netF* was not tested. Finally, incomplete clinical and antimicrobial-exposure data precluded disease-association and risk-factor analyses. These limitations define the scope of interpretation without diminishing the study’s value as multiregional baseline evidence.

## 5. Conclusions

This study provides the first multiregional assessment from Türkiye to examine *cpa*-confirmed *C. perfringens* together with *C. difficile*-specific DNA and toxin-gene targets in archived fecal samples from the same foal population. *Clostridium perfringens* was confirmed in 16.7% of samples, and the examined isolates displayed a *cpa*-only profile among the genes investigated, compatible with toxin type A within the limits of the panel. The *C. difficile*-specific *tpi* target was detected in 4.0% of samples, and *tpi*, *tcdA*, and *tcdB* were co-detected in 2.3%. Both bacterial targets occurred in samples from all five provinces; these geographic observations are descriptive and do not establish equal distribution.

The results represent sample-level baseline data from archived material. Molecular detection of *tpi*, *tcdA*, and *tcdB* does not establish viable *C. difficile*, toxin production, or clinical infection, and the available clinical data do not permit disease-association analysis. Prospective studies combining standardized clinical information, quantitative bacteriology, *C. difficile* culture, toxin detection, and broader virulence profiling—including *netF*—are warranted.

## Figures and Tables

**Table 1:** Oligonucleotide primers and molecular targets used for culture confirmation and toxin-gene profiling of *Clostridium perfringens* and PCR detection of *Clostridioides difficile*-specific DNA and toxin-gene targets.

Organism	Target Gene	Primer Sequence (5′–3′)	Amplicon (bp)	Reference
*C. difficile*	*tpi*	F: AAAGAAGCTACTAAGGGTACAAA; R: CATAATATTGGGTCTATTCCTAC	230	[6,13]
*C. difficile*	*tcdA*	F: AGATTCCTATATTTACATGACAATAT; R: GTATCAGGCATAAAGTAATATACTTT	369	[6,13]
*C. difficile*	*tcdB*	F: GGAAAAGAGAATGGTTTTATTAA; R: ATCTTTAGTTATAACTTTGACATCTTT	160	[6,13]
*C. perfringens*	*cpa*	F: GTTGTAAGCGCAGGACATGTTAAG; R: CATGTAGTCATCTGTTCCAGCATC	402	[14]
*C. perfringens*	*cpb*	F: GCGAATATGCTGAATCATCTA; R: GCAGGAACATTAGTATATCTTC	196	[14,18]
*C. perfringens*	*cpb2*	F: AGATTTTAAATATGATCCTAACC; R: CAATACCCTTCACCAAATACTC	567	[14]
*C. perfringens*	*etx*	F: CGGGTGATATCCATCTATTC; R: CCACTTACTTGTCCTACTAAC	655	[14,18]
*C. perfringens*	*iap*	F: AAACGCATTAAAGCTCACACC; R: GTGCATAACCTGGAATGGCT	293	[14,18]
*C. perfringens*	*cpe*	F: GGAGATGGTTGGATATTAGG; R: GACAGGGGCATACCCATATA	506	[14]
*C. perfringens*	*netB*	F: GCTGGTGCTGGAATAAATGC; R: TCGCCATTGAGTAGTTTCCC	384	[14,19]

Note: bp, base pairs; F, forward primer; R, reverse primer. References in the rightmost column identify the sources used for the primer sequences and/or assay procedures.

**Table 2:** Province-level distribution of culture and molecular findings.

*tpi*/*tcdA*/*tcdB* Co-Detection, *n* (%) [95% CI]	*C. difficile Tpi*-Positive, *n* (%) [95% CI]	*cpa*-Confirmed *C. perfringens*, *n* (%) [95% CI]	Presumptive *C. perfringens*, *n* (%)	Samples Examined	Province
1 (4.3) [0.8–21.0]	1 (4.3) [0.8–21.0]	4 (17.4) [7.0–37.1]	5 (21.7)	23	Malatya
2 (4.5) [1.3–15.1]	2 (4.5) [1.3–15.1]	7 (15.9) [7.9–29.4]	8 (18.2)	44	Şanlıurfa
1 (1.4) [0.2–7.6]	2 (2.8) [0.8–9.7]	12 (16.9) [9.9–27.3]	12 (16.9)	71	Bursa
2 (2.0) [0.6–7.0]	5 (5.0) [2.2–11.2]	17 (17.0) [10.9–25.5]	18 (18.0)	100	Eskişehir
1 (1.6) [0.3–8.6]	2 (3.2) [0.9–11.0]	10 (16.1) [9.0–27.2]	10 (16.1)	62	İzmit/Kocaeli
7 (2.3) [1.1–4.7]	12 (4.0) [2.3–6.9]	50 (16.7) [12.9–21.3]	53 (17.7)	300	Total

Percentages use the number of samples examined in each row as the denominator. Confidence intervals are two-sided 95% Wilson intervals. Geographic estimates are descriptive and exploratory.

## Data Availability

The original contributions presented in this study are included in the article. Further inquiries can be directed to the corresponding author.
